# Perceived Experiences and Needs of Digital Resources Among Postpartum Women in the United Arab Emirates: Qualitative Focus Group Study

**DOI:** 10.2196/53720

**Published:** 2024-12-16

**Authors:** Nivine Hanach, Roba Saqan, Hadia Radwan, Wegdan Baniissa, Nanne de Vries

**Affiliations:** 1 Faculty of Health, Medicine, and Life Sciences, Care and Public Health Research Institute Maastricht University Maastricht Netherlands; 2 Health Promotion Group Research Institute of Medical and Health Sciences University of Sharjah Sharjah United Arab Emirates; 3 Department of Clinical Nutrition and Dietetics, College of Health Sciences, Research Institute of Medical and Health Sciences University of Sharjah Sharjah United Arab Emirates; 4 Department of Nursing, College of Health Sciences, Research Institute of Medical and Health Sciences University of Sharjah Sharjah United Arab Emirates

**Keywords:** digital health, social support, telemedicine, postpartum women, focus group, maternal health, postpartum mental health, postpartum depression, emotional support, health information

## Abstract

**Background:**

The postpartum period is a critical phase in a woman's life, marked by various physical, psychological, and social challenges. In light of the rapid proliferation and uptake of digital technologies, particularly in the United Arab Emirates (UAE), mothers increasingly seek informational and emotional support from digital resources. No previous study has thoroughly explored the usage of various digital resources beyond telehealth services in the UAE. This literature gap is particularly relevant for the postpartum period, which remains largely understudied in the UAE.

**Objective:**

This study aims to delve into the digital experiences of postpartum women in the UAE by exploring the types of resources they navigate and the purposes those resources serve. In addition, it seeks to identify their perspectives and needs regarding digital resources that support their postpartum journey.

**Methods:**

Four focus groups were conducted synchronously on the web, involving a total of 27 multicultural mothers (mean age 32.47, SD 4.56 years), between 2 and 12 months post partum and living in the UAE. Descriptive interpretive thematic analysis was used to analyze the data.

**Results:**

Sixteen out of 27 women exhibited severe depressive symptoms at the time of the discussions (Edinburgh Postnatal Depression Scale score of >12). Two main themes were generated from the analysis: (1) Mothers’ Experiences with Digital Resources: Participants valued digital resources for providing immediate information, convenience, and support. They primarily used these resources to seek information on infant health, parenting advice, and emotional support through web-based communities. However, the abundance of conflicting information and the pressure to conform to health recommendations often created stress and anxiety. (2) The Perceived Need for Digital Resources: Despite their extensive use of digital resources, mothers articulated the need for a reliable UAE government digital platform tailored specifically to postpartum care, offering trusted information on infant health and postpartum mental well-being. They also emphasized the need for tailored postpartum telemedicine services and moderated web-based discussion forums to foster peer support among mothers.

**Conclusions:**

This study reveals the multifaceted role of digital resources in supporting mothers during the postpartum period, highlighting unmet needs that present opportunities for advancing postpartum care in the UAE. It demonstrates the importance of developing reliable digital solutions for postpartum women, especially regarding mental health and to enhance access to care through tailored telemedicine services. Collaborative efforts are required to ensure the implementation of user-centered digital platforms. Future research should focus on the diverse needs of postpartum women, including cultural sensitivity, the feasibility of telemedicine services, and the integration of partner support in digital interventions to improve maternal health outcomes.

## Introduction

The postpartum period is a critical phase in a woman’s life and is characterized by a multitude of physical, psychological, and social challenges [1]. As they adapt and adjust to the demands of this transitional period, mothers may experience ambivalence, anxiety, and depression [2,3]. Previous research has found that receiving sufficient postpartum social support improves maternal and child outcomes [4,5]. Traditionally, mothers would turn to their families, friends, and health care providers for informational and emotional support [6]. When mothers perceive that social support is more readily available, they have greater self-efficacy in their competence to meet the needs of their children [7]. In light of the rapid proliferation and uptake of digital technologies, studies have shown that digital resources are an important growing source of immediate and concise information as well as reassurance and support for postpartum mothers [8,9]. These resources include social media platforms, websites, mobile health (mHealth) apps, short message services, and emails, which may also encompass telemedicine services—the remote administration of health care to patients via telecommunication technologies [10-12].

The integration of digital technologies into postpartum care offers several practical advantages. These include facilitating the understanding of health information presented in different formats, improving connectivity, increasing health care accessibility, and ensuring convenient and timely support for mothers after childbirth [13]. Importantly, digital health care technologies could therefore mitigate geographic obstacles and disparities in health care access, particularly for low-income postpartum women [12,13]. Nevertheless, existing evidence reveals that while digital health may offer a novel opportunity for postpartum women to address their concerns, it also brings about certain drawbacks and considerations. Postpartum women often encounter an overwhelming volume of conflicting information from diverse digital platforms, causing confusion and increased anxiety [14]. Another critical consideration is the digital literacy skills of postpartum women. Effective usage of digital resources relies on the ability to navigate and assess web-based health information accurately to make informed decisions [6]. Moreover, prior studies examining the digital technology needs of postpartum women consistently uncovered new mothers’ desire to have improved streamlined communication with their health care providers, a local community with other mothers of similar experiences, and personalized digital features to ensure tailored and relevant resources [15,16].

The United Arab Emirates (UAE) is at the forefront when it comes to the use of digital technologies in the Middle East. In 2024, there were 10.14 million web users in the UAE, representing 99% of the total population [17]. Specifically, the number of mobile phone subscribers with web access reached 9.55 million out of more than 22 million mobile subscribers [17,18].

Telehealth is defined as the delivery and facilitation of health and health-related services via telecommunications and digital communication technologies [10]. A recent study demonstrated that the pandemic has substantially accelerated the adoption of telehealth services in the UAE, with a notable preference for usage among women [19]. Conversely, Ahmed et al [20] revealed remarkable disparities in the usability of telehealth technologies, using information and communication technologies, particularly among Arab and non-Arab women. Arab women exhibited lower levels of usability across all the usability subscales. This implies that the distinctive diversity of the UAE population [21] further adds complexity to the usage of digital technologies and emphasizes the requirement for a comprehensive examination of users’ experiences and perceptions to cater digital technologies to their specific needs and cultural norms.

Furthermore, to the best of our knowledge, no previous study has thoroughly explored the usage of various digital resources beyond telehealth services in the UAE. This gap in the literature is particularly relevant for the postpartum period, which remains largely understudied in the UAE and extends to the relatively unexplored realm of usage patterns and needs for digital resources among postpartum women. Therefore, this study aims to delve into the digital experiences of postpartum women in the UAE, including the types of resources navigated and the purposes they serve, in addition to identifying their perspectives and needs for digital resources during the postpartum period. This could ensure alignment between the development of targeted digital interventions and the needs of postpartum women, which in turn could contribute to the enhancement of postpartum care in the digital age.

## Methods

### Study Design

This study is part of an exploratory, multipurpose exploratory, descriptive focus group study conducted between September 2022 and January 2023. Its primary aim is to comprehensively investigate various aspects of postpartum experiences among mothers in the UAE. Specifically, in this study, we delve into the usage of digital resources and the perceived needs of postpartum women. The choice of an exploratory focus group design is supported by previous research, indicating its suitability for addressing sensitive topics targeting mothers [22] and establishing an understanding of the phenomena of interest [23,24]. Furthermore, the study conforms to Krueger and Casey’s [25] guidelines for designing and developing focus group interviews and adheres to the established COREQ (Consolidated Criteria for Reporting Qualitative Research) [26]. In order to bolster the credibility and reliability of the findings of the studies, researchers have adopted a reflexive approach to the research process, as described by Dodgson [27].

### Ethical Considerations

The ethical committee for research ethics at the University of Sharjah approved this study (REC-22-08-29). Written informed consent was obtained from all participants prior to the commencement of the study. Measures were implemented to ensure participant confidentiality, including anonymizing data and securely storing all audio recordings and transcripts. Participants did not receive compensation or direct benefits for their involvement in the study.

### Study Setting and Sampling

In this study, participants were purposively recruited from diverse sources including the maternity wards of 2 hospitals in Dubai and Sharjah, where potential participants were initially approached by a nurse after childbirth. Eligible women who showed interest in the study and agreed to be referred to the research team were then informed about the study’s objectives, timeline, and process of their involvement by the study researchers. Upon their consent, recruitment was confirmed. Other recruitment sources included social media platforms, such as Facebook and Instagram, where targeted posts and advertisements were used to reach potential participants. In addition, web-based communities and parenting forums that cater to mothers served as platforms for sharing information about the study, allowing for direct engagement with interested participants. Word-of-mouth referrals from participating mothers further facilitated recruitment, and those interested were invited to attend the focus group discussions, thereby ensuring a diverse range of perspectives and experiences were represented.

### Participants

Women aged 18-45 years, who had given birth within the last year, and were either UAE citizens or residents of other nationalities were eligible for participation. Women who gave birth prematurely (before 37 weeks of gestation) or whose newborns required intensive care throughout the research period were excluded. There was no restriction on whether the mother had a singleton or multiple pregnancies to establish a thorough understanding of the various postpartum experiences. The study’s researchers outlined the study’s aims, objectives, and intended involvement process to eligible women before distributing the participant information sheet and written consent form. Three out of 30 women withdrew from the study due to availability and time conflicts. The participants’ demographics and characteristics are shown in Table 1.

**Table 1:** The sociodemographic characteristics of postpartum women in the United Arab Emirates from the 4 focus group discussions.

Parameters			Values
Focus group 1, n			6
Focus group 2, n			8
Focus group 3, n			6
Focus group 4, n			7
**EPDS^a^ score, n (%)**			
	Normal (<9)	8 (29.6)	
	Moderate depressive symptoms (10-12)	3 (11.1)	
	Severe depressive symptoms (>12)	16 (59.3)	
Age (years), mean (SD)			32.47 (4.56)
**Parity, n (%)**			
	Primiparous	14 (51.9)	
	Multiparous	13 (48.1)	
	Number of siblings, median (range)	1 (0-3)	
**Nationality, n (%)**			
	Arab	14 (51.9)	
	Western	9 (33.3)	
	South Asian	4 (14.8)	
**Expatriate, n (%)**			
	Yes	24 (88.9)	
	No	3 (11.1)	
**Highest education level, n (%)**			
	High school	3 (11.1)	
	Technical diploma	2 (7.4)	
	University degree	22 (81.5)	
**Employment status, n (%)**			
	Not employed	9 (33.3)	
	Part-time	3 (11.1)	
	Full-time	12 (44.4)	
	Self-employed	3 (11.1)	
**Maternity leave** **, n (%)**			
	Not employed	9 (33.3)	
	<3 months	10 (37)	
	≥3 months	8 (29.6)	
**Husband’s employment status, n (%)**			
	Not employed	1 (3.7)	
	Full-time	23 (85.1)	
	Part-time	—^b^	
	Self-employed	3 (11.1)	
**Husband’s education level** **, n (%)**			
	High school	1 (3.7)	
	Technical diploma	2 (7.4)	
	University degree	24 (88.9)	
**Family income, n (%)**			
	5000 to <10,000 AED (US $1361 to <$2722)	2 (7.4)	
	10,000 to 15,000 AED (US $2722 to $4087)	—^b^	
	>15,000 AED (>US $4087)	25 (92.5)	
**Perception of family income, n (%)**			
	Not enough	3 (11.1)	
	Enough	16 (59.3)	
	More than enough	8 (29.6)	
**Infant sex, n (%)**			
	Boy	15 (55.5)	
	Girl	10 (37)	
	Twin girls	2 (7.4)	
**Childbirth type, n (%)**			
	Vaginal	8 (29.6)	
	Cesarean	19 (70.4)	
**Current feeding practices, n (%)**			
	Breastfeeding	6 (22.2)	
	Formula feeding	14 (51.8)	
	Both	7 (26)	
**Having a nanny, n (%)**			
	Yes	16 (59.3)	
	No	11 (40.7)	
**Having a house helper, n (%)**			
	Yes	19 (70.3)	
	No	8 (29.6)	
**History of psychological disorder, n (%)**			
	No	23 (85.2)	
	Yes	4 (14.8)	
**History of depression, n (%)**			
	No	18 (66.7)	
	Yes	9 (33.3)	
**Complications during childbirth, n (%)**			
	No	17 (63)	
	Yes	10 (37)	
**Complications during pregnancy, n (%)**			
	Breech presentation	2 (7.4)	
	Prelabor rupture of membranes	2 (7.4)	
	Intrauterine growth restriction	1 (3.7)	
	Gestational diabetes mellitus	3 (11.1)	
	None	19 (70.4)	
**Relationship with husband, n (%)**			
	Poor	2 (7.4)	
	Average	8 (29.6)	
	Excellent	17 (63)	
**Primary childcare support, n (%)**			
	Father	8 (29.6)	
	Family	3 (11.1)	
	Nanny	16 (59.3)	
**Husband support, n (%)**			
	Rarely	5 (18.5)	
	Sometimes	6 (22.2)	
	Usually	5 (18.5)	
	Always	11 (40.7)	

^a^EPDS: Edinburgh Postnatal Depression Scale.

^b^Not available.

### Processes

#### Data Collection

A web-based questionnaire via Google Forms was completed by the participants to obtain an overview of their characteristics including sociodemographic information (eg, age, nationality, education, employment status, financial status, and marital status), obstetric data (type of delivery, complications during pregnancy and childbirth, infant sex, and feeding practices), history of medical and psychological conditions, perceived relationship with the husband, and husband’s support during the postpartum period. To identify women exhibiting postpartum depression symptomatology, the questionnaire also included the Edinburgh Postnatal Depression Scale (EPDS) [28]. Each item is assessed on a 4-point scale with a range of 0-3, giving a total score between 0 and 30. Higher scores indicate increased depression symptomatology. A cutoff point of >12 identifies women with depression symptomatology; however, a cutoff point of >9 has been suggested to increase sensitivity for the purposes of community screening [29]. A validated Arabic version of the EPDS with adequate psychometric properties [30] was used with Arabic-speaking participants. To mitigate any potential labeling harm, mothers with high EPDS scores were assured of the nondiagnostic nature of the measure [31], and they were encouraged to seek further assessment in clinical settings, if needed. In addition, it was emphasized that confidentiality is rigorously upheld at all times, and depression scores are neither disclosed nor discussed in the focus groups.

#### Focus Group Interviews

In accordance with the preference of participating mothers owing to their schedule constraints, the focus group interviews were hosted through a computer video interface using Zoom Software (Zoom Video Communications). Four focus groups were conducted, 2 of which comprised 6 women each, while the remaining groups consisted of 8 and 7 women, respectively. Depending on the number of participants and the extent of discussion required to comprehensively address the emerging topics, the length of the focus group interviews ranged between 90 and 120 minutes. All participants were informed that the interviews would be video recorded before participating in the discussions. In addition, participants were informed that they had the right to withdraw from the study at any time or to omit any of the interviewer’s questions without any negative consequences or penalty. Each session was initiated with the facilitator, who has extensive experience in qualitative research involving postpartum women (NH, PhD, female) reviewing the informed consent form and outlining the discussion’s ground rules. The facilitator emphasized the importance of each participant’s comfort by affirming that there is no “right” or “wrong” response and that all viewpoints are valid. In each focus group, the facilitator (NH) introduced the cofacilitator, an experienced researcher (RS, MSc, female), and acknowledged their roles in the study. The facilitator and cofacilitator were from different cultural backgrounds who maintained neutrality throughout the discussions. This was intended to encourage participants to share their experiences and perspectives openly. Participants were informed that the discussions and data collection were kept strictly confidential and used solely for research purposes. The language spoken in the group discussions was English, as it is extensively used and understood in the UAE, although Arab participants were advised to opt for Arabic if they felt more comfortable expressing themselves in it (however, none did). Based on the existing literature, the research team (NH, NdV, HR, RS, and WBI) collectively developed a semistructured interview guide that covered a series of questions that transitioned from open questions about the mothers’ experiences and use of digital resources to more specific questions about their perceived need for digital resources during the postpartum period (Multimedia Appendix 1). Furthermore, the facilitator used probing questions and reflecting statements to deepen the discussion and clarify the participants’ responses such as “Could you further explain? Can you tell me more about this experience? Can you give me an example? Let me repeat what you have said.” The facilitator then gave a short summary of key points that had been discussed after each focus group meeting. Before concluding the session, participants were invited to share any additional thoughts and encouraged to ask questions. A concurrent analysis was undertaken, and data collection continued until saturation was achieved and no new themes were identified [32].

### Data Analysis

Descriptive quantitative analysis (frequency count and percentages) was performed using Statistical Package for the Social Sciences software (version 26.0; IBM Corp). The discussions were recorded and professionally transcribed verbatim, with additional context provided by field notes taken by the cofacilitator. Thematic analysis was chosen as the method of analysis for the focus groups because of its versatility in accommodating both inductive (data-driven) and deductive (theory-driven) approaches [33]. This allowed the identification of both anticipated and unanticipated themes. Six phases of Braun and Clarke’s [34] reflective thematic analysis were used. Two researchers (NH and RS) independently analyzed and developed codes and themes. They read the transcripts multiple times and familiarized themselves with the data. A color-coding method was then applied within ATLAS.ti (version 23; ATLAS.ti Scientific Software Development GmbH) to identify distinct concepts and patterns and form initial codes. Related codes were collated to generate preliminary themes and subthemes and then reviewed by a third researcher (NdV). Any discrepancies were resolved through discussions and consensus (systematic debriefings) among all the study researchers (NH, NdV, HR, WBI, and RS), who were from different cultural backgrounds, ages, sexes, and parity status. By integrating diverse perspectives and interpretations, this has facilitated rigorous and thorough analysis of the data, which in turn has increased the reliability and validity of the study findings. The themes were then refined, defined, and tabulated. The final phase involved writing the report, which provided further clarity for each theme.

### Member Checking

To ensure the dependability and transferability of the results, member checking was carried out. After the completion of thematic analysis, each participant was given an opportunity to review the derived themes and subthemes and provide additional insights. All the participants corroborated the findings, which were found to be robust and representative of their experiences; no objections or refutations were raised.

## Results

Twenty-seven women, who were between 2 and 12 months post partum took part in 1 of the 4 focus group discussions. Overall, the majority (16/27, 59.2%) exhibited severe depressive symptoms. The demographic and descriptive data for the participating women are outlined in Table 1. Two main themes were identified: (1) mothers’ experience with the use of digital resources and (2) the perceived need for digital resources. A thematic map presenting the main themes, subthemes, and participants’ insights was derived from the thematic analysis (Multimedia Appendix 2).

### Mothers’ Experience With the Use of Digital Resources

All participants in the study reported using at least one of the following digital resources during the postpartum period: Google search engine, mobile apps, government websites, and social media platforms. The mothers valued the inherent convenience offered by these digital resources in terms of accessibility and timeliness. A key finding of the focus group discussions was the use of these digital resources to seek information related to parenting and infant concerns or emotional support and validation in web-based communities.

### Digital Information–Seeking for Parenting and Infant Concerns

The majority of mothers reported using the Google search engine to obtain immediate health information related to parenting or infant health, especially when they were worried and had limited access to health care providers, such as in the middle of the night. However, some mothers deliberately avoided using the search engine because it provided general advice rather than tailored recommendations and reported having had previous negative experiences that resulted in unnecessary stress and anxiety.

[…] What should I do? Where should I start? I googled. And then it showed that my son could be autistic, but it turned out to be a simple developmental delay and now he is completely fine. It put me under a lot of stress and that’s why I stopped googling.

Focus group 3

Some multiparous mothers expressed that they did not search for as much information as when they had their first child as they perceived themselves to be more experienced.

With my second son I don't even read honestly, I don't even need. It's a totally different journey, I don't know, like we get more confident with being a mom, for example I know what's happening, I know that it is a phase and it's gonna pass.

Focus group 1

Some expatriate mothers reported the frequent use of government-funded educational websites for parenting and infant health information. Examples of these were the government-sponsored Australian parenting website “Raising Children” and the United Kingdom’s National Health Service. They described these websites as noncommercial owing to their provision of credible information by experts about various stages of infant development and infant health. They praised the reliability and trustworthiness of the information on these websites.

I haven't lived in Australia for almost 15 years, but there's like a website called Raising children, a government sponsored health site that I feel familiar with and trust its information.

Focus group 2

Most mothers also reported the use of mobile apps for tracking developmental milestones, newborn sleep and routine, and receiving advice about the overall infant’s health. Some of the highlighted mobile apps were “Little ones,” “wonder week,” what to expect,” and “BabyCenter.” These apps were valued for their instantaneous access to information, user-friendly interface, and personalized features such as the ability to log key details about the infant’s sleep, feedings, and daily activities.

You get immediate information, and you can log your baby’s routine so it indirectly helped my mental health in the sense that it helped me stay organized.

Focus group 1

Mothers also turned to social media platforms to seek parenting and infant-related information, with Instagram and TikTok being particularly popular choices. Notably, some mothers indicated that they exclusively followed the accounts of experts and health care providers to ensure access to accurate information, especially from those who shared similar advice as their personal health care providers (eg, pediatricians). Their rationale for this approach was to establish trust in the content they consumed. Conversely, other mothers disclosed that they selectively followed accounts of mothers or experts who aligned with a “similar philosophy of motherhood.” They expressed concerns that conflicting viewpoints could significantly affect their mental health by either creating or exacerbating anxiety.

Good pediatricians get on TikTok and since every video is about 30 seconds long, they provide quick pieces of information about your baby. You're like, “Oh my God, that makes sense.” So, what happens is that when I realize that a specific person, I’m following shares a lot of views with, my daughter’s pediatrician, then I start to build trust with them, and I begin to listen closely…not following blindly, but listening closely.

Focus group 4

Across all group discussions, participants consistently recognized the potential usefulness of digital resources in accessing regular information. However, they also voiced feelings of overwhelming uncertainty, particularly when discrepancies emerged in shared information on different digital resources. Some mothers further expressed feelings of stress, guilt, disappointment, and a sense of inadequacy when they could not meet the health recommendations, such as exclusive breastfeeding, infant sleep patterns, and routine. Mothers expressed amplified concerns about their infants not meeting expected developmental milestones. They felt pressured to adhere strictly to institutional guidelines, especially first-time mothers. On the other hand, some mothers took a cautious stance by critically evaluating web-based information and relying primarily on their health care providers for guidance.

### Emotional Support and Validation in Web-Based Communities

Parenting mobile apps were deemed particularly helpful when they included chat rooms or discussion forums with other mothers who shared similar interests. In these forums, mothers could exchange their experiences and seek advice, if needed. The ability to maintain anonymity within these discussions was highly valued by mothers, as it increased their comfort level and facilitated disclosure of their own experiences. Even mothers who passively consumed information without engaging in discussions found web-based forums to be highly beneficial.

And even sometimes I literally go on the app when I need to confirm something, I read the discussions because it's just a group of moms who gave birth around the same time as I did. So, I find that pretty helpful.

Focus group 4

One mother reported having joined a paid web-based mother’s academy platform led by a clinical dietitian and lactation consultant, which she described as beneficial as it not only provided credible information but also included a mother’s forum where they could share their infant feeding experiences, and the health care provider would address any of their concerns. Other mothers reported using Facebook to establish connections and develop social friendships by joining groups of mothers who also shared similar characteristics, such as mothers of twins in Dubai, Australian mothers, and British mothers. This was represented as a form of dealing with isolation and a lack of social support, especially among expatriate mothers who have no family or friends living nearby to reach out to.

I joined Facebook groups because I don’t have friends here and it has been good because it's people that just come from the same kind of mindset and culture as me and we understand each other.

Focus group 1

When mothers were asked about the common topics or areas of interest discussed on these mothers’ forums, they mentioned breastfeeding, infant routines, sleeping patterns, postpartum mental health, and occasionally, sensitive personal topics.

### The Perceived Need for Digital Resources

Mothers were asked about their perceived need for digital resources in terms of the type, platforms, content, and potential interactive features. The group discussions shed light on the preferences and priorities of various digital tools for supporting their postpartum journey.

### Government-Led Digital Resources

Across the 4 focus group discussions, mothers expressed their need to have a well-established UAE governmental digital platform, in the form of either a website or a mobile app, that could serve as a reliable source of information to support their decision to care for their infant.

Educational information related to child development and health, so I can use that information to justify my decision to people around me.

Focus group 2

Mothers who shared their experiences with the use of government-backed websites in their respective countries were asked about the specific utilities offered by these websites. A key feature of these resources is their accessibility and trustworthiness. They valued the credibility of information, knowing that it had been reviewed by medical experts. In addition, these mothers highlighted the use of the web-based symptom checker. They clarified that this service offers guidance, rather than formal medical diagnoses, for individuals experiencing any symptoms and directs them to a health adviser as needed. Although not explicitly tailored to postpartum mothers, this service was perceived as highly beneficial as it could address and alleviate concerns related to infant health commonly experienced by mothers during the postpartum period. Therefore, mothers emphasized the importance of implementing a comparable governmental digital platform for postpartum care within the UAE, specifically tailored to meet the unique needs of postpartum women.

When mothers were probed about the specific information they are intrigued to have access to on such hypothetical governmental websites during the postpartum period, they emphasized the need for educational material in the form of concise articles addressing topics related to their own mental well-being, offering guidance on adapting to changes in the postpartum period, and promoting self-care, especially when experiencing “self-identity crises.” Mothers notably expressed the desire to gain knowledge about when and where to seek mental health support in the postpartum period.

More awareness about mental health and what a mother goes through during the postpartum period, when things might be more alarming when you need to seek support? Professional Support?

Focus group 3

### Postpartum Telemedicine Services

Mothers mentioned additional digital features and interactive components as imperative to support their postpartum experience. The ability to have remote access to a health care provider, particularly via web-based consultation, came up often across all group discussions. Some mothers expressed that their ideal preference would be to have a “*one-stop-shop*” mobile app with access to information and an embedded virtual consultation service with a UAE health care provider. They further declared that having this option can save their transportation time to the clinic, especially for minor inquiries when no physical examination is needed. One mother pointed out that her child’s pediatrician is frequently fully booked and difficult to reach, making virtual consultations extremely valuable whenever she has health-related questions. Some mothers’ desire for a virtual consultation with a health care provider stems from the fact that this service has been highly promoted during the COVID-19 pandemic. However, these mothers conveyed their aspiration for the extension of such virtual health care accessibility for postpartum women.

Because of living in Dubai, we have learned something from the online world in COVID which was truly helpful. Being at home and having the ability to access doctors by phone or even working from home which made life more convenient because sometimes we simply don’t have the time.

Focus group 4

Time is incredibly valuable here; we are constantly juggling many tasks. Saving even just 30 minutes can make a difference. For example, I calculate that it takes me 30 minutes to get to the doctor and another 30 minutes to return, so that’s an hour spent on travel alone. So, it is two hours and in two hours I can accomplish a lot.

Focus group 3

Furthermore, some mothers suggested that having a live chat with a health care provider offering immediate responses to mothers’ concerns and urgent inquiries would support their postpartum journey and ease their worry, especially during nighttime hours when access to health care services can be challenging. Others proposed that for general inquiries that do not require an instant response, an “ask the expert” forum where they post their questions and receive reliable answers from a health care provider would be extremely beneficial.

The ability to ask a question, not in a one-on-one consultation, potentially in like a Q&A, a question that you’re struggling to find an answer for, to actually have it answered by a professional.

Focus group 1

Mothers expressed a preference for virtual access to various health care providers, including pediatricians, lactation consultants, clinical dietitians, child psychologists, speech therapists, and occupational therapists. The demand for lactation consultants arose from the challenges many mothers faced in receiving adequate breastfeeding support and finding local lactation consultation services. Mothers highlighted the importance of having access to clinical dietitians to assist with infant feeding practices such as breastfeeding and complementary feeding, as well as postpartum weight management. In addition, mothers frequently mentioned their desire to have access to a therapist who specializes in postpartum mental health. Given that some mothers perceived therapy to be relatively expensive in the UAE, they underlined the cost-effectiveness of such access.

Going and seeking out therapy is like, so difficult. It’s expensive, first of all, but also like, it is just like the last thing on your mind when you have a new baby. So, I think that would be really important.

Focus group 4

Some mothers also noted that having access to a therapist through a mobile app could surmount cultural barriers and stigma associated with seeking mental health support. Others suggested the creation of a digital directory of UAE mental health professionals who specialize in maternal mental health, in case virtual consultation is not a feasible service, as they often find it difficult to locate specialized therapists. To enhance the usability of such a directory, mothers recommended including the cultural backgrounds of the therapists, which would facilitate finding a therapist whose cultural background aligns with their own.

I won’t be judged if I am speaking with a therapist because they might not even know.

Focus group 4

### Web-Based Discussion Forums With Other Mothers

The need for web-based discussion forums with other mothers was frequently mentioned in all 4 focus group discussions. These forums were valued for maintaining a sense of connection and fostering a community of peer support among mothers. They explained that having access to a group of mothers stratified by interest or characteristics such as infant age, feeding practices, cultural background, and so forth, is comforting and offers rewarding emotional support. One mother articulated a preference for joining a group of experienced mothers instead of reaching out to a health care provider via a web-based consultation, as she believed that a health care provider is not always “*emotionally supportive*” particularly when something contradicts the guidelines. She further elaborates by giving the example of cosleeping.

I’ll give you an example. Okay, so, I co-sleep with my baby, he will not sleep in any other way. And I have to sleep with him literally, a pediatrician will tell you that’s wrong, it’s against safe sleep, like all these things, right? pediatricians don’t believe in that kind of stuff. Only another mom who is co-sleeping her child would truly understand.

Focus group 4

Conversely, 2 mothers expressed reservations about mothers’ groups, such as chat rooms or forums, highlighting some potential drawbacks. They mentioned that these web-based communities often disseminate misleading information and unsolicited advice, which can sometimes lead to a competition among mothers.

Talking with other mothers in places like chat rooms or forums ends up with a lot of spiel and competition talk. It's kind of like what you see on Instagram, you know? They are not experts.

Focus group 1

## Discussion

### Principal Findings

This study aimed to explore the digital experiences of postpartum women in the UAE and identify their needs for digital resources during the postpartum period. The findings revealed that mothers valued digital resources for providing immediate information, convenience, and support. They used them to seek infant health information and parenting advice and to obtain emotional support through web-based communities. In addition, mothers expressed the need for a reliable UAE government digital platform tailored to postpartum care, offering trusted information on infant health and postpartum mental well-being. They also emphasized the importance of accessing web-based discussion forums with other mothers to foster peer support and the need for postpartum telemedicine services.

Consistent with previous qualitative studies [35], the need for information is prominent during the first year following childbirth. Our findings demonstrate that mothers’ primary use of digital resources is to access information related to infant care and infant health. This is particularly pertinent among primiparous women, who may perceive a greater lack of information than multiparous women, whose need for information appears to decrease and become more specific for subsequent children [8]. In addition, Slomian et al [36] revealed that mothers tend to turn to the web for information significantly more frequently during the postpartum period than during pregnancy. It is hypothesized that pregnant women initially seek and discuss information with their midwives and gynecologists. This discrepancy in information-seeking behavior implies a potential gap in the provision of information by health care providers throughout the antenatal period [36].

Socioeconomic status is asserted to play a moderating role in the use of digital resources. Higher socioeconomic status is associated with greater access to web-based health information [37]. Conversely, Guerra-Reyes et al [38] found that low-income postpartum women rely on web-based sources to obtain self-care and infant health information. However, a particular concern arises in the form of the digital divide, which may also occur because of lower digital literacy in lower-educated populations [39]. Postpartum women with lower educational levels tend to seek reassuring health information, whereas those with higher educational levels tend to exhibit more discernible web-based information-seeking behaviors by searching for trustworthy information on accredited and authoritative websites [38]. The latter could also explain the extensive need of our participating mothers, who mainly had high levels of education, for trustworthy and credible information led by government authorities or health professionals. However, it is important to acknowledge that when the need for information in response to a health concern is perceived as pressing, mothers become less concerned about misinformation by merely searching for validation or guidance, irrespective of their education level [6]. Therefore, promoting science and health literacy should be prioritized to empower postpartum women with the necessary skills to effectively navigate evidence-based web-based health information and make informed health decisions.

While the mothers in this study emphasized the need for informational support during the postpartum period, they equally described their need for emotional, affirmational, and social network support. To address these needs, mothers’ support-seeking behaviors expanded to web-based communities and support groups facilitated by various digital platforms, including mobile apps’ postpartum forums and Facebook groups. Peer support is defined as the exchange of resources and experiences between like-minded peers with similar experiences [40]. Accessibility to web-based peer support groups is deemed particularly helpful for mothers when navigating the increased demands of infant care after childbirth [41]. Besides offering flexibility, web-based support groups can empower mothers by fostering a sense of normalcy and competence, improving their feelings of self-determination, and promoting social engagement [42,43]. Although the mothers in this study highly valued these virtual support groups, they also drew attention to potential negative aspects, including the dissemination of inaccurate information, misleading advice, and intense arguments. Accordingly, Xie et al [43] suggested that midwives, trained on communication and social skills, moderate web-based parenting communities with the aim of providing both informational and emotional support. Nonetheless, despite the “drama,” mothers perceive that the benefits of these web-based forums outweigh the disadvantages [41]. However, it is worth noting that further research is needed to determine effective strategies to moderate and enhance the quality of virtual mother groups.

During group discussions, as mothers elaborated on their use of digital resources during the postpartum period, they highlighted their extensive access to information on infant care and health. Interestingly, when the conversation shifted toward their perceived need for digital resources, mothers expressed a paucity of information on postpartum mental health. They conveyed their desire to acquire sufficient knowledge on this topic, including guidance on when and how to seek local professional support. These insights were similarly shared by mothers with postpartum depression, who identified a knowledge gap regarding postpartum depression and available care options [15]. Given that the majority of women in our study also exhibited severe depressive symptoms (EPDS >12) at the time of discussion, this could potentially demonstrate the unmet needs of postpartum women in terms of their mental health. The lack of knowledge and understanding of postpartum mental health, as well as attitudes and beliefs surrounding treatment, has been consistently linked to stigma, which poses a significant barrier to mothers’ help-seeking behavior [44]. A counterargument to the previously uncovered observations is that numerous eHealth and mHealth resources are available on the web for postpartum mental health. While this assertion holds partial validity, the underlying issue pertains primarily to the quality of web-based information on postpartum mental health. An assessment of 14 smartphone apps using the Silberg Scale revealed an average score of 3.0 (SD 1.52) out of 9 points, indicating poor information quality about postpartum depression and anxiety [45]. Similarly, when more than 20 postpartum mental health websites were evaluated, the vast majority scored low to moderate on the DISCERN tool [46,47]. This was attributed to the absence of information sources and insufficiently detailed descriptions of postpartum mental health treatment options and their benefits [47]. Furthermore, low actionability scores indicate a lack of focus on providing tools and clear instructions for supporting action-taking and informed decision-making among postpartum women [46]. Therefore, it is imperative to consider the psychoeducational needs of postpartum women when designing and developing digital interventions that focus on usability and information quality.

In this study, virtual access to health care professionals was underlined as a prominent digital need among postpartum women. This finding is not surprising in the context of the UAE, where telehealth services have gained substantial recognition and rapid adoption, particularly since the onset of the COVID-19 pandemic [48]. Recent UAE studies have evaluated the usability of telemedicine consultations and patient perceptions in the general population, revealing high satisfaction levels [20,49,50]. However, significant disparities in satisfaction levels emerged when different demographic and contextual factors were considered. For instance, non-Arab participants reported higher levels of satisfaction than Arab participants in terms of the usefulness, ease, and effectiveness of teleconsultations [20]. In addition, specific factors, such as sex, education, frequency of use, delivery format, and teleconsultation duration, contribute to variations in patient satisfaction [20,49,50]. When comparative survey research was conducted in the United States among postpartum women regarding the use and quality of virtual care following childbirth, more than 80% (184/231) of postpartum women reported a high quality of care defined as convenient, easy, safe, and with good information [51]. However, specific concerns have been highlighted that impact their satisfaction, including feeling less connected to their providers, not getting the same level of care as they would in person [51,52], and the unavailability of their providers to discuss postpartum depression, lactation challenges, and contraception [53]. Interestingly, this could be a plausible explanation for our findings, where mothers frequently emphasized the need for virtual access to specialized therapists and lactation consultants, but none mentioned the need for access to obstetrician-gynecologists after childbirth. Unfortunately, this situation parallels that in actual care, as prior research has shown that mothers often experience disappointment and dissatisfaction with their health care providers in addressing their postpartum mental health and emotional needs, causing them to refrain from discussing their concerns [54]. In the postpartum period, virtual consultations can improve continuity of care, lactation support, and counseling on contraception, in addition to screening and treatment for mental health problems [55,56]. To date, no telemedicine interventions have been specifically designed for postpartum care in the UAE. Therefore, it is essential to implement and evaluate telemedicine interventions that address the specific needs of mothers and account for potential cultural and demographic disparities to ensure equitable access and satisfaction and, in turn, optimize the delivery of virtual care in the country.

### Strengths and Limitations

To the best of our knowledge, this study represents the first investigation in the UAE to explore the digital needs of postpartum women. Furthermore, this study serves as a continuation of a qualitative inquiry into the perceived mental health experiences and needs of mothers after childbirth. By using synchronous web-based focus group discussions via videoconferencing, we were able to ensure geographic accessibility and recruit participants from various emirates, including Sharjah, Dubai, and Abu Dhabi. This inclusiveness allows for a comprehensive understanding of diverse experiences, considering the variations in health care systems across these regions. The web-based format was convenient and flexible for the participating mothers, allowing them to engage from their preferred locations and even breastfeed their infants during discussions. Such consideration of participant comfort and uninterrupted participation enhances the credibility and dependability of the study’s findings.

Several limitations should also be addressed when interpreting the findings of the study. First, the analysis incorporated the perceptions of mothers who were healthy with no medical complications (eg, cardiovascular disease and diabetes), who were married, who were mostly of a high sociodemographic status in terms of education and income, and who gave birth to full-term infants. Therefore, representativeness for all mothers in the UAE cannot be claimed. Second, a significant limitation of this study is the restricted diversity among the enrolled participants. For instance, women of South Asian ethnicity were underrepresented (4/27, 14.8%), despite South Asians comprising 59.4% of the UAE population [57]. In addition, the mothers’ unique needs were not disentangled according to the defined postnatal stages (ie, early, intermediate, and late post partum) and severity of postpartum depression symptomatology, which could have influenced their interactions with various digital resources. Another limitation is that the majority of the participants exhibited severe depressive symptoms, potentially leading to different digital resource needs compared with those with no depressive symptoms, thus limiting the generalizability of the findings.

### Conclusions, Recommendations, and Implications

This study provided in-depth insights into the experiences and needs of mothers using digital resources during the postpartum period. It also highlights the multifaceted role of digital resources in supporting mothers’ postpartum journeys. Postpartum women described a multitude of unmet needs, which subsequently present opportunities to bridge gaps and address these requirements. Of primary interest is the development and implementation of a dedicated digital platform for postpartum care by the UAE government, which aims to improve access to accurate and reliable information, especially concerning postpartum mental health. Collective efforts from health care providers, researchers, science communicators, and policy makers are essential to ensure the design and development of evidence-based content and user-centered digital platforms that align with the preferences and usability requirements of mothers. The study also suggests that future research should investigate the needs of postpartum women from diverse ethnic and cultural backgrounds to develop digital platforms that are not only culturally sensitive but also linguistically adapted, thus ensuring their relevance and effectiveness in serving the UAE’s heterogeneous population. In addition, future research should evaluate the digital needs of a more diverse sample of postpartum women to ensure comprehensive and inclusive postpartum digital resources, including those who have experienced preterm birth, have medical conditions, and are socioeconomically disadvantaged. Furthermore, given the evident demand for postpartum telemedicine services, it is imperative to evaluate the feasibility, cost-utility, and cost-effectiveness of offering virtual consultations to postpartum women. This could help estimate the long-term use of telemedicine services and their benefits for maternal health and infant outcomes. Finally, with the significant protective effect of partner support against postpartum mental health problems [58], future studies should investigate the effectiveness of integrating both postpartum women and their partners within digital interventions as a means to enhance partner education and support.

## Appendix

Multimedia Appendix 1 Focus group interview guide.

Multimedia Appendix 2 A thematic map presenting the main themes, subthemes, and participants' insights derived from the thematic analysis.

## Abbreviations

COREQ: Consolidated Criteria for Reporting Qualitative Research
EPDS: Edinburgh Postnatal Depression Scale
mHealth: mobile health
UAE: United Arab Emirates

## Data Availability

The datasets generated during and/or analyzed during this study are available from the corresponding author on reasonable request. Data will be available in the form of deidentified transcripts.

## References

[1] Paladine HL, Blenning CE, Strangas Y (2019). Postpartum care: an approach to the fourth trimester. Am Fam Physician.

[2] Lupton D (2016). The use and value of digital media for information about pregnancy and early motherhood: a focus group study. BMC Pregnancy Childbirth.

[3] Jaks R, Baumann I, Juvalta S, Dratva J (2019). Parental digital health information seeking behavior in Switzerland: a cross-sectional study. BMC Public Health.

[4] Sufredini F, Catling C, Zugai J, Chang S (2022). The effects of social support on depression and anxiety in the perinatal period: a mixed-methods systematic review. J Affect Disord.

[5] White LK, Kornfield SL, Himes MM, Forkpa M, Waller R, Njoroge WFM, Barzilay R, Chaiyachati BH, Burris HH, Duncan AF, Seidlitz J, Parish-Morris J, Elovitz MA, Gur RE (2023). The impact of postpartum social support on postpartum mental health outcomes during the COVID-19 pandemic. Arch Womens Ment Health.

[6] Donelle L, Hall J, Hiebert B, Jackson K, Stoyanovich E, LaChance J, Facca D (2021). Investigation of digital technology use in the transition to parenting: qualitative study. JMIR Pediatr Parent.

[7] Chang YE (2017). Pathways from mothers' early social support to children's language development at age 3. Infant Child Dev.

[8] Slomian J, Bruyère O, Reginster JY, Emonts P (2017). The internet as a source of information used by women after childbirth to meet their need for information: a web-based survey. Midwifery.

[9] Moon RY, Mathews A, Oden R, Carlin R (2019). Mothers' perceptions of the internet and social media as sources of parenting and health information: qualitative study. J Med Internet Res.

[10] (2018). What is telehealth?. NEJM Catal.

[11] Eijkelboom MCLC, Kalee MM, de Kleijn RAMR, van Wijngaarden JJJ, de Jonge RRR, van der Schaaf MFM, Frenkel JJ (2022). Making knowledge clips with patients: what learning mechanisms are triggered in medical students?. Patient Educ Couns.

[12] Schnitman G, Wang T, Kundu S, Turkdogan S, Gotlieb R, How J, Gotlieb W (2022). The role of digital patient education in maternal health: a systematic review. Patient Educ Couns.

[13] Lau Y, Wong SH, Cheng LJ, Lau ST (2023). Exploring experiences and needs of perinatal women in digital healthcare: a meta-ethnography of qualitative evidence. Int J Med Inform.

[14] Price SL, Aston M, Monaghan J, Sim M, Tomblin Murphy G, Etowa J, Pickles M, Hunter A, Little V (2018). Maternal knowing and social networks: understanding first-time mothers' search for information and support through online and offline social networks. Qual Health Res.

[15] Lackie ME, Parrilla JS, Lavery BM, Kennedy AL, Ryan D, Shulman B, Brotto LA (2021). Digital health needs of women with postpartum depression: focus group study. J Med Internet Res.

[16] Zingg A, Carter L, Rogith D, Franklin A, Selvaraj S, Refuerzo J, Myneni S (2021). Digital technology needs in maternal mental health: a qualitative inquiry. Stud Health Technol Inform.

[17] (2024). Official Global Media Insights. UAE internet Stat.

[18] (2024). Open data sets. Telecommunications and Digital Government Regulatory Authority.

[19] Swidan A, Alnoon NA, Makki I, Zidan M, Alhammadi H, Rahmani N, Alameeri A, Al Falasi A, Fakhroo A, Al Mulla J, Al Marzooqi L (2022). Effect of COVID-19 pandemic on patient utilization of the telemedicine services in Dubai. Dubai Med J.

[20] Ahmed F, Zabin RG, Abukoush RS, Alnahoum AA, Alkhawaldeh MY, Al-Yateem N, Subu MA, Alboloshi AM, Marzouqi AA (2023). Usability differences of telehealth technologies by multi-linguistic and multi-cultural users in UAE.

[21] (2024). Fact sheet. The Official Portal of the UAE Government.

[22] Skelton K, Evans R, LaChenaye J, Amsbary J, Wingate M, Talbott L (2018). Utilization of online focus groups to include mothers: a use-case design, reflection, and recommendations. Digit Health.

[23] Stebbins RA (2001). Exploratory Research in the Social Sciences.

[24] Sandelowski M (2010). What's in a name? Qualitative description revisited. Res Nurs Health.

[25] Krueger RA, Casey MA (2002). Designing and Conducting Focus Group Interviews.

[26] Tong A, Sainsbury P, Craig J (2007). Consolidated Criteria for Reporting Qualitative Research (COREQ): a 32-item checklist for interviews and focus groups. Int J Qual Health Care.

[27] Dodgson JE (2019). Reflexivity in qualitative research. J Hum Lact.

[28] Cox JL, Holden JM, Sagovsky R (1987). Detection of postnatal depression. Development of the 10-item Edinburgh postnatal depression scale. Br J Psychiatry.

[29] Murray L, Carothers AD (1990). The validation of the edinburgh post-natal depression scale on a community sample. Br J Psychiatry.

[30] Abou-Saleh MT, Ghubash R (1997). The prevalence of early postpartum psychiatric morbidity in Dubai: a transcultural perspective. Acta Psychiatr Scand.

[31] Sheehan AM, McGee H (2013). Screening for depression in medical research: ethical challenges and recommendations. BMC Med Ethics.

[32] Glaser BG, Strauss A (1967). The Discovery of Grounded Theory. Strategies for Qualitative Research.

[33] Boyatzis RE (1998). Transforming qualitative information. Thematic Analysis and Code Development.

[34] Braun V, Clarke V (2022). Conceptual and design thinking for thematic analysis. Qual Psychol.

[35] Finlayson K, Crossland N, Bonet M, Downe S (2020). What matters to women in the postnatal period: a meta-synthesis of qualitative studies. PLoS One.

[36] Slomian J, Reginster J, Emonts P, Bruyère O (2021). Identifying maternal needs following childbirth: comparison between pregnant women and recent mothers. BMC Pregnancy Childbirth.

[37] Nölke L, Mensing M, Krämer A, Hornberg C (2015). Sociodemographic and health-(care-)related characteristics of online health information seekers: a cross-sectional German study. BMC Public Health.

[38] Guerra-Reyes L, Christie VM, Prabhakar A, Harris AL, Siek KA (2016). Postpartum health information seeking using mobile phones: experiences of low-income mothers. Matern Child Health J.

[39] van Deursen A, van Dijk J (2010). Internet skills and the digital divide. New Media Soc.

[40] Shalaby RAH, Agyapong VIO (2020). Peer support in mental health: literature review. JMIR Ment Health.

[41] Teaford D, McNiesh S, Goyal D (2019). New mothers' experiences with online postpartum forums. MCN Am J Matern Child Nurs.

[42] Bîră M, Buzoianu C, Tudorie G (2020). Social support mediated by technology. A netnographic study of an online community for mothers. Rom J Commun Public Relat.

[43] Xie J, He Z, Burnett G, Cheng Y (2021). How do mothers exchange parenting-related information in online communities? A meta-synthesis. Comput Hum Behav.

[44] Jones A (2019). Help seeking in the perinatal period: a review of barriers and facilitators. Soc Work Public Health.

[45] Zhang MW, Ho RC, Loh A, Wing T, Wynne O, Chan SWC, Car J, Fung DSS (2017). Current status of postnatal depression smartphone applications available on application stores: an information quality analysis. BMJ Open.

[46] Kirby PL, Reynolds KA, Walker JR, Furer P, Pryor TAM (2018). Evaluating the quality of perinatal anxiety information available online. Arch Womens Ment Health.

[47] Hardman MP, Reynolds KA, Petty SK, Pryor TAM, Pierce SK, Bernstein MT, Furer P (2022). An evaluation of the quality of online perinatal depression information. BMC Pregnancy Childbirth.

[48] (2023). Telemedicine. The Official Portal of the UAE Government.

[49] Al-Sharif GA, Almulla AA, AlMerashi E, Alqutami R, Almoosa M, Hegazi MZ, Otaki F, Ho SB (2021). Telehealth to the rescue during COVID-19: a convergent mixed methods study investigating patients' perception. Front Public Health.

[50] Al Meslamani AZ, Aldulaymi R, El Sharu H, Alwarawrah Z, Ibrahim OM, Al Mazrouei N (2022). The patterns and determinants of telemedicine use during the COVID-19 crisis: a nationwide study. J Am Pharm Assoc (2003).

[51] Marshall C, Gutierrez S, Hecht H, Logan R, Kerns J, Diamond-Smith N (2023). Quality of prenatal and postpartum telehealth visits during COVID-19 and preferences for future care. AJOG Glob Rep.

[52] Sullivan MW, Kanbergs AN, Burdette ER, Silberman J, Dolisca S, Scarry J, Soffer M, Kaimal A, Bryant Mantha A, Bernstein SN (2022). Acceptability of virtual prenatal care: thinking beyond the pandemic. J Matern Fetal Neonatal Med.

[53] Davis A, Bradley D (2024). Telemedicine utilization and perceived quality of virtual care among pregnant and postpartum women during the COVID-19 pandemic. J Telemed Telecare.

[54] Nan Y, Zhang J, Nisar A, Huo L, Yang L, Yin J, Wang D, Rahman A, Gao Y, Li X (2020). Professional support during the postpartum period: primiparous mothers' views on professional services and their expectations, and barriers to utilizing professional help. BMC Pregnancy Childbirth.

[55] Hawkins SS (2023). Telehealth in the prenatal and postpartum periods. J Obstet Gynecol Neonatal Nurs.

[56] Hanach N, de Vries N, Radwan H, Bissani N (2021). The effectiveness of telemedicine interventions, delivered exclusively during the postnatal period, on postpartum depression in mothers without history or existing mental disorders: a systematic review and meta-analysis. Midwifery.

[57] (2023). United Arab Emirates-Country Summary. Central Intelligence Agency.

[58] Zorer PB, Akbulut ST, Dirik G (2019). Role of attachment patterns and partner support in postpartum depression. Psikiyatr Guncel Yaklasimlar.

