# Supplementary material for: Perceived Experiences and Needs of Digital Resources Among Postpartum Women in the United Arab Emirates: Qualitative Focus Group Study
Source: J Med Internet Res. 2024 Dec 16;26:e53720. doi: 10.2196/53720 (PMC11694600; doi:10.2196/53720)
Supplement: Multimedia Appendix 1 [file jmir_v26i1e53720_app1.docx]

**Focus group interview guide**

1. **Welcome**

Hello moms, my name is ….., I am a PhD candidate at Maastricht University in the Netherlands. I will be moderating today’s focus group session alongside …., who will be assisting, taking notes and handling the logistics of this session.

1. **Introduction**

First, I would like to start by thanking each one of you for taking the time to participate in this research. I greatly appreciate your cooperation. The main objectives of this session are to understand the mothers’ challenges during the postpartum period and to further explore their perceived needs to support their mental health.

You were invited because you are a mother who lives in the UAE and have recently given birth.

I would like to inform you all that this focus group session will be recorded because we don't want to miss any of your valuable comments and to allow for an accurate analysis of the themes that will emerge along the way. You may say very important things in the discussion and we can't write fast enough to get them all down.

We will be on a first name basis today, and we won't include any of your names in any of the write-ups or data analysis that occur in this project. So, please rest assured that this discussion is only for research purposes and it is strictly confidential.

Before we begin, there are some ground rules I would like to go over to ensure a respectful and free-flowing conversation:

1. Only one person may speak at a time. This is important for the usefulness of the recording of the session.
2. Please ensure that any cell phones are off or silenced, unless absolutely necessary. If you are required to take a phone call, or have a side conversation, please mute your microphone.
3. You do not have to give a response for every question; however, I would like to hear from each of you at some point in our discussions and will try to make sure that everyone receives a chance to speak. Also, you are free to answer whether in Arabic or English.
4. There are no “right” or “wrong” answers, all your opinions are valid. We should all respect each other’s’ opinions and experiences, so please say whatever is true for you without fear of judgement.

Before we move on, do you have any question?

Ice breaker:

Well, let’s begin. Let’s get to know each of other. Tell me, how many children do you have and for how long have you been living in the UAE?

1. **Questions**

Now let’s speak about the postpartum period,

1. How do you describe the postpartum period in 2 to 3 words?
2. What emotions did you or do you currently experience after giving birth?

Tell me more, what do you mean…

if we want to discuss the challenges of the postpartum period,

1. Based on your experience, what are the difficulties or challenges you faced or currently face during the postpartum period?
2. What kind of help did you need the most?
3. who helped you or currently help you the most after giving birth?

🡪 How? What do you mean? Tell me more about it?

1. What are the things that mostly affected your mental health after you gave birth?

🡪 tell me more about it; do you have anything to add; what do you mean by…?

Speaking of mental health,

1. how much do you think you need mental health support after giving birth?

🡪 Tell me more about it? What do you mean?

1. How do you think each of your partner/ family/ friends (social resources) can support your mental health?
2. What about your healthcare provider, how do you think healthcare providers can support your mental health?
3. What form of support did you or do you currently seek to improve your mental health?
4. What did you do you do or currently do to improve your mental health after giving birth?

Now let’s speak about the use of online resources after childbirth.

1. Tell us about your experience with using digital resources during the postpartum period?
   1. What are some of the online resources have you used?
   2. What kind of content were you looking for? Why?
   3. How do you feel that these online resources have supported or impacted your postpartum journey?
2. In your opinion, what kind of digital resources would be most beneficial for supporting your postpartum journey?
   1. What particular interactive components do you think would be most useful in these digital resources?

Tell me more, what do you mean…

1. **Closing**

(Read back a summary of what has been discussed.)

Is there anything else would you like to add to the topic?

Does anyone have any final questions, comments, or concerns?

We have come to the end of our session today. I want to thank you all for your honest opinions and input on this topic. This is an extremely important stage of this project, and you were extremely helpful in providing useful feedback.
