# Supplementary figures and images for: Perceived Experiences and Needs of Digital Resources Among Postpartum Women in the United Arab Emirates: Qualitative Focus Group Study
Source: J Med Internet Res. 2024 Dec 16;26:e53720. doi: 10.2196/53720 (PMC11694600; doi:10.2196/53720)

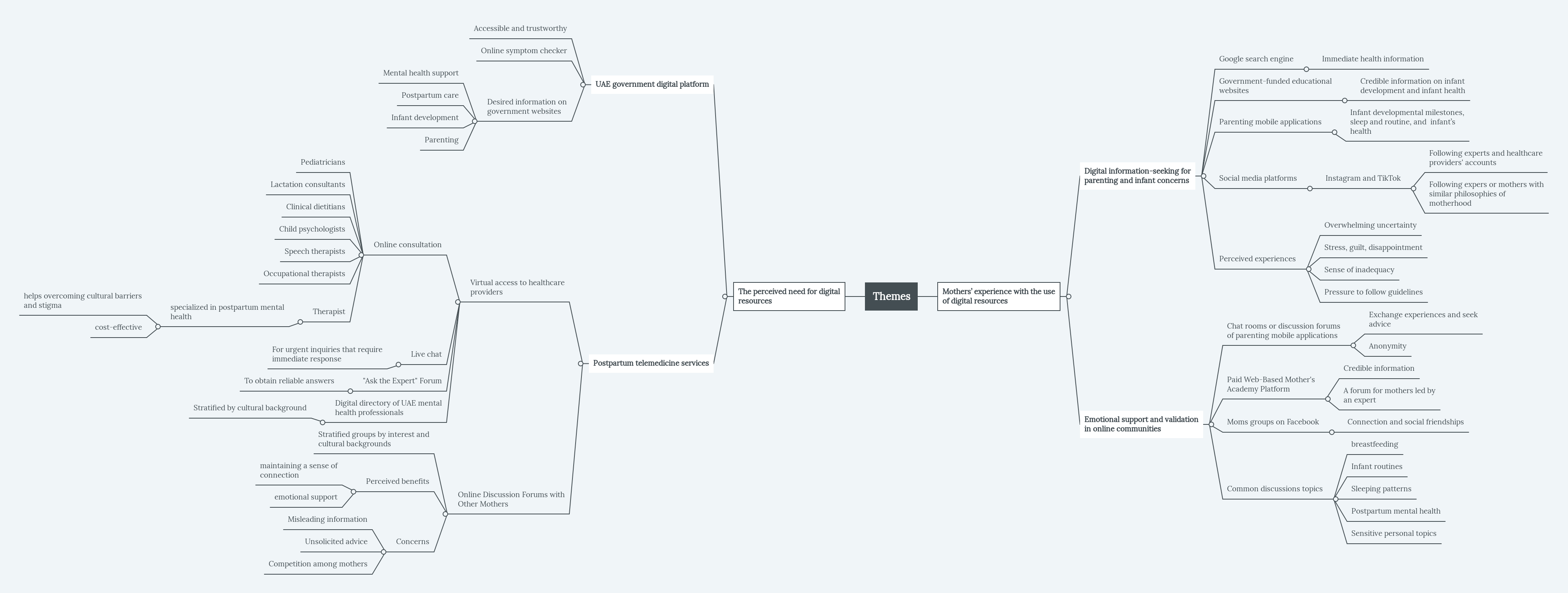

Supplement: Multimedia Appendix 2 [file jmir_v26i1e53720_app2.png]
